# EUS-Guided Combined Injection Therapy as a Secondary Prophylaxis of Gastric Variceal Bleeding in a Patient Contraindicated for TIPS: Case Report

**DOI:** 10.3390/medicina60010116

**Published:** 2024-01-08

**Authors:** Krasimir Asenov, Rosen Dimov, Maria Kraeva, Yordanka Basheva-Kraeva

**Affiliations:** 1 Section “Gastroenterology”, Second Department of Internal Diseases, Medical Faculty, Medical University—Plovdiv, 4000 Plovdiv, Bulgaria; asenov.krasimir@abv.bg; 2 Gastroenterology Clinic, University Hospital “Kaspela”, 4000 Plovdiv, Bulgaria; 3 Department of Special Surgery, Medical Faculty, Medical University—Plovdiv, 4000 Plovdiv, Bulgaria; rosendimov68@gmail.com; 4 Surgical Department, University Hospital “Kaspela”, 4000 Plovdiv, Bulgaria; 5 Department of Otorhynolaryngology, Medical Faculty, Medical University—Plovdiv, 4000 Plovdiv, Bulgaria; 6 Department of Ophtalmology, University hospital “St. George”, 4000 Plovdiv, Bulgaria

**Keywords:** coils, cyanoacrylate, embolization, endoscopic ultrasound, gastric varices

## Abstract

*Background*: Although bleeding from gastric varices is less observed than esophageal variceal bleeding (VB) (25% vs. 64%), it is associated with an exceedingly high mortality rate of up to 45%. Current guidelines suggest that endoscopic cyanoacrylate injection therapy (ECI) is the first-line treatment for gastric variceal bleeding (GVB). A major concern, however, is the possibility of embolic incidents, which are clinically evident in approximately 1% of cases. There are no guidelines for secondary prophylaxis of GVB. Radiological treatments using a transjugular intrahepatic portosystemic shunt (TIPS) or balloon occlusive transvenous obliteration (BRTO) are considered viable. However, they are not universally inapplicable; for instance, in the setting of pulmonary hypertension (TIPS). EUS-guided combined injection therapy (EUS-CIT) (embolization coils + cyanoacrylate) is an emerging procedure with a perceived reduced risk of systemic embolization. *Case presentation*: A patient with alcoholic liver cirrhosis was subjected to EUS-CIT as a secondary prophylaxis for GVB. He had three VB episodes of prior presentation treated by endoscopic band ligation (EBL) and ECI. Due to recurrent episodes of bleeding, he was referred to TIPS, but was considered contraindicated due to severe pulmonary hypertension. EUS-CIT was conducted with two embolization coils inserted into the varix, followed by an injection of 1.5 mL of cyanoacrylate glue. A 19 Ga needle, 0.035″ 14/70 mm coils, non-diluted n-butyl-caynoacrylate, and a transgastric approach were utilized. There were no immediate complications. Complete obliteration of the GV was observed in a follow-up endoscopy on day 30. Subsequent endoscopies in months three and six showed no progression of gastric varices. *Conclusions*: Our initial experience with EUS-CIT suggests that it can be successfully used as secondary prophylaxis for recurrent GVB.

## 1. Introduction 

Variceal bleeding (VB) is a common complication of liver cirrhosis with an annual incidence of 8–10% [1]. In long-term follow-ups, VB occurs in about 40% of compensated liver cirrhosis and up to 85% of decompensated liver cirrhosis patients [2]. While recently published guidelines and research indicate a gradual transition to non-invasive modalities for evaluating various gastrointestinal disorders, including gastro-oesophageal varices (GOV), endoscopy is still the cornerstone in managing VB [3,4,5,6]. 

Gastric varices (GV) represent a complex collection of vascular shunts between the porto-splenic venous system and the systemic veins of the abdomen and thorax. GV is uncommon (17–24% in patients with portal hypertension) compared to esophageal varices (EVs) (up to 85%). Sarin et al. established that bleeding from GV is rarer than EVs (25% vs. 64%, respectively) [7]. However, gastric variceal bleeding (GVB) is more severe and associated with higher mortality (45% vs. 20%, respectively) [7]. Sarin’s classification of gastric varices is the most commonly used system (Table 1). 

Recent guidelines suggest that in GOV type 1, endoscopic band ligation (EBL) or endoscopic cyanoacrylate injection (ECI) are viable options [8,9,10]. In GOV type II and IGV type I, ECI is the preferred treatment modality [8,9,10]. 

The major drawback of ECI therapy is the potential for fatal adverse events (AEs). ECI complication rates range from 7% to as high as 20% [11]. The risk of glue detachment and subsequent embolism is a source of grave concern and manifests clinically in about 1% of cases, likely occurring in a significantly higher proportion of patients. 

Endoscopic ultrasound (EUS) has become increasingly popular over the last two decades for treating various pancreatico-biliary disorders [12,13]. Until recently, its usage for vascular endotherapy had been overlooked. To reduce the incidence of embolic complications associated with glue injection therapy, Binmoeller et al. introduced an EUS-guided management of gastric varices combining coil and cyanoacrylate injections [14]. While still in its infancy and not universally adopted, initial studies have shown improved clinical outcomes compared to conventional ECI in terms of AEs (10% vs. 20%, systemic embolism being exceedingly rare), rebleeding rate (0% vs. 38%), and the need for reintervention (10% vs. 60%) [11]. 

According to existing guidelines, there is a lack of high-quality data to suggest optimal therapeutic approaches for secondary prophylaxis of GVB. It is noted that transjugular intrahepatic portosystemic shunt (TIPS) and balloon occlusive retrograde transvenous obliteration (BRTO) probably perform similarly in cases of refractory bleeding [9].

In the current paper, we present a case of EUS-guided combined injection therapy (EUS-CIT) (coils + n-butyl-cyanoacrylate) for the secondary prophylaxis of gastric variceal bleeding in a patient with three previous bleeding episodes treated with EBL and ECI and was ineligible for TIPS due to severe pulmonary hypertension. 

## 2. Case Description

The patient was a 65-year-old male with alcoholic cirrhosis. The diagnosis was established 4 years prior to the current presentation, and he abstained from alcohol consumption thereafter. Comorbidities included arterial hypertension, established 15 years prior to the initial diagnosis of liver cirrhosis for which he received regular therapy with angiotensin receptor blockers (Telmisartan 10 mg/day). In the past year, he experienced three episodes of variceal bleeding. Initially, he underwent an episode of hematemesis. Urgent upper endoscopy within six hours of symptoms onset revealed esophageal and variceal bleeding. EBL immediately controlled the bleeding. Two units of blood were transfused and the patient was discharged on post-procedure day four. No gastric varices were described at that point. An endoscopic follow-up was recommended within 20 days, but he refused. Non-selective beta-blockers (Propranolol 20 mg twice a day) were prescribed.

The patient was admitted again for hematemesis and melena 45 days later. Gastroscopy found no bleeding in the esophagus, but upon stomach examination, a type II GOV with a tortuous course and verices of about 2 cm was established. The bleeding spot was visualized. ECI was performed with 2 mL of N-butyl cyanocrylate injected under endoscopic guidance. The bleeding was immediately controlled. A transfusion of three units of blood was necessary to achieve a hemoglobin level of 93 g/L. He was discharged without any therapy modifications.

About two months later, the patient underwent another episode of upper gastrointestinal bleeding. Although I upper endoscopy revealed no signs of active bleeding, the presence of fresh blood in the stomach and a red spot on one of the fundal varices was observed. Endoscopic treatment was not commenced. Considering the refractory character of the bleeding, the patient’s eligibility for interventional treatment through TIPS was decided and he was referred to a tertiary center.

Pre-procedural echocardiography, however, showed severe pulmonary hypertension with a mean pulmonary artery pressure of >45 mmHg. Thus, he was considered contraindicated for TIPS. He was prescribed Tadalafil 10 mg twice daily and referred for reevaluation after 45 days. Follow-up echocardiography failed to establish any sonographic signs of improvement. At this point, he was referred to our center to discuss alternative therapeutic approaches.

### 2.1. Pre-Procedure Evaluation

At admission, the patient had not experienced any episodes of overt gastrointestinal bleeding in the last two months. Ultrasonography established typical cirrhotic transformation of the liver parenchyma (enlarged liver—craniocaudal diameter up to 200 mm, irregular contours, heterogenous structure, lobe disproportion) and an enlarged spleen (160/80 mm). Portal vein evaluation showed mild dilation up to 12 mm. Doppler ultrasound assessment was not performed due to a perceived low correlation with severe portal hypertension. There were only mild ascites established sonographically. Lab tests showed Hb—93 g/L, platelets—38 000 mm^3^, albumin—29 g/L, bilirubin—36.2 µmol/L, and an international normalized ratio (INR) of 1.36. There was no alteration in electrolytes or creatinine levels. Based on the clinical and lab findings, the patient was classified as class B according to the Child–Turcotte–Pugh classification (see Appendix A, Table A1) and had a model of end-stage liver disease (MELD-Na) score of 13.

An upper endoscopy was performed, which established grade II esophageal varices (Baveno classification, see Appendix A, Table A2) and large, type I GOV, tortuous varices measuring about 1.5 cm with red spots. Based on these endoscopic findings, the patient was considered at high risk of recurrent bleeding. Considering concomitant pulmonary hypertension, an increased risk of potentially fatal embolic AEs was perceived. It was then decided that EUS-CIT (coil + cyanoacrylate) might be safer than conventional ECI for avoiding thromboembolism.

A planned intervention was thoroughly explained to the patient, with all possible outcomes, AEs, and viable alternatives diligently described. Written and oral informed consent was obtained before the procedure.

The impaired coagulation was concerning so an adequate correction was planned. A total of 500 mL of fresh frozen plasma was transfused the night before the procedure. A total of 12 units of thrombocyte concentrate were also transfused immediately before and during the intervention. Prophylactic antibiotic treatment with 2 g of Ceftriaxone/d intravenously was initiated. A proton pump inhibitor was not prescribed. The patient was advised to continue his routine therapy (Telmisartan, Tadalafil, Propranolol).

### 2.2. Technique Description

This procedure was executed under general anesthesia with tracheal intubation using a combination of fentanyl, midazolam, sevoflurane, suxamethonium (Lysthenon), atracurium besylate (Tracrium), and propofol. 

The patient was placed in a left lateral position. A curvilinear echoendoscope (Olympus GF-UCT180, Olympus, Hamburg, Germany), combined with a Hitachi-Aloka ProSound Alpha 7 (Hitachi, Tokyo, Japan) ultrasound device, was introduced and positioned at the level of the cardia. Insufflation with CO_2_ was used instead of ambient air (Olympus UCR, Hamburg, Germany). A total of 200 mL of 0.9% sodium chloride was installed in the stomach to improve acoustic coupling. Once at the level of the cardia, the procedure was performed under sonographic guidance. Gastric varices were visualized sonographically, and a Doppler ultrasound was performed to confirm the presence of blood flow. The widest diameter of the varix was 10 mm. We aimed to embolize the vessels located under the mucosa and not the perforated vessel (despite being easily identified) due to the perceived risk of embolization. Although a transcrural approach was intended, the position of the endoscope was inadequate, so a transgastric puncture was attempted. 

Once an adequate position was achieved sonographically, the varix was punctured with a 19 Ga fine needle aspiration (FNA) needle (Expect^TM^ needle; Boston Scientific, Marlborough, MA, USA). As anticipated, a transgastric approach was associated with a more angulated position of the tip of the endoscope, leading to a more difficult puncture of the target vessel and insertion of the coils. Blood was aspirated to verify an adequate position, followed by rinsing the needle with 4 mL of 5% glucose solution. Next, two 0.035 inch 14 mm/7 cm embolization coils (Nester Embolization coil; Cook Medical, Bloomington, IN, USA) were introduced into the varix. The technique consisted of inserting the coils into the FNA needle, and then pushing them out into the varix using a needle stylet. The procedure was executed under combined ultrasound and fluoroscopic guidance using Philips BV Pulsera C-arm (Philips, Best, The Netherlands). Although primarily used as a scaffold for subsequent glue injection, an immediate reduction of blood flow in the varix was observed upon coil deployment.

Following coil insertion, another aspiration of blood and rinsing with glucose solution were executed to reconfirm the position prior to glue injection. Eventually, 1.5 mL of pure (not mixed with Lipiodol) N-2-butyl cyanoacrylate was injected into the varix. To compensate for the volume retained in the needle, the cyanoacrylate syringe was filled before application with 1 mL more cyanoacrylate than intended for injection. The needle was withdrawn from the vessel but not from the endoscope to minimize the risk of endoscope damage. The controlled Doppler examination showed that blood flow was almost absent from the target vessels.

At this point, the endoscopic reevaluation showed moderate amounts of blood in the fundus of the stomach, but no active bleeding. Gentle probing with a cannula was performed, with the varices established to have a hard consistency.

**Figure 1:**
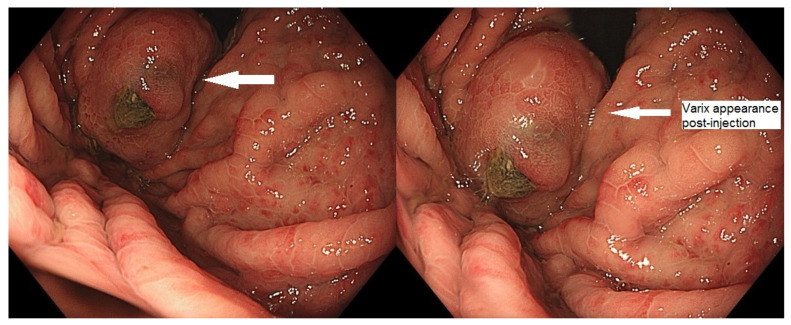
Endoscopic appearance of gastric varix post-embolization.

**Figure 2:**
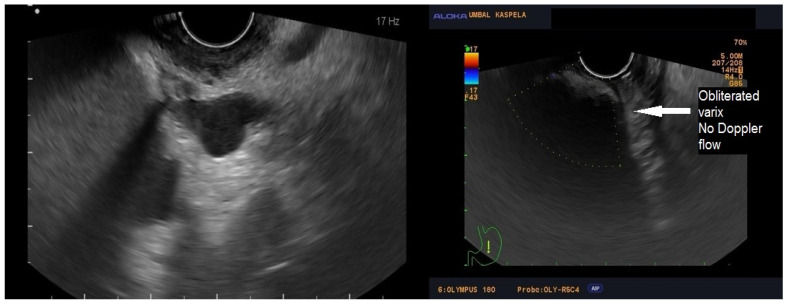
Ultrasonography showing absent Doppler flow in the treated vessel.

**Figure 3:**
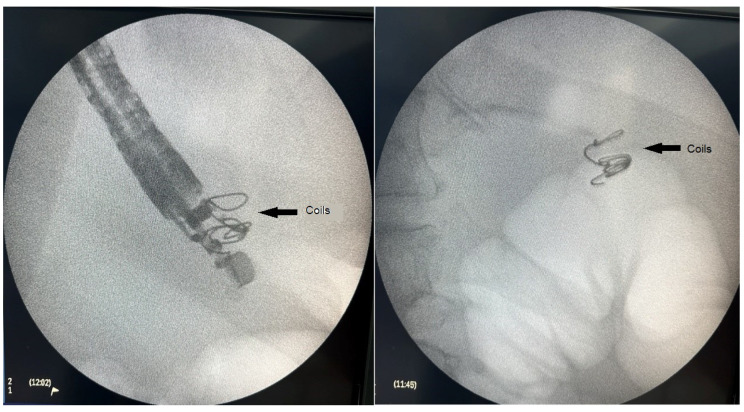
Fluoroscopic image of the introduced embolization coils.

Figure 1, Figure 2 and Figure 3 present post-procedural endoscopic, endosonographic, and fluoroscopic findings.

### 2.3. Post-Procedure

The post-procedure period was uneventful. The patient restored their fluid intake the same day, a semi-solid diet the day after, and was discharged on post-procedure day two. Lab tests showed only a minor decrease in hemoglobin to 86 g/L. No vasoactive medications or proton pump inhibitors were prescribed. An antibiotic prophylaxis was administered until discharge. 

A follow-up endoscopy was performed 30 days after the intervention. The Doppler EUS showed no blood flow in the gastric varices. According to the existing guidelines, EBL (Super7; Boston Scientific, Marlborough, MA, USA) of EVs was electively performed to achieve eradication. Upper endoscopies were performed at 3 and 6 months without progression signs of GOV.

The patient has not experienced any bleeding episodes at nine months post-procedure.

A summary of the disease course is provided in Table 2.

## 3. Discussion

The current case delineates some of the risk factors for GVB. Our patient experienced two prior episodes of GVB. The presence of red spots, decompensated liver cirrhosis, and the location and size (>10 mm) of the varices (GOV type II) confirmed the patient’s exceedingly high risk of recurrent hemorrhage. An interesting observation was the lack of gastric varices at the initial presentation. This finding correlates with Sarin et al.’s study in which GV develops in about 9% of patients following obliteration of EVs [7]. 

The current standard of care for GVB is ECI. Existing data suggest that ECI is superior to alternative methods of hemostasis (ethanol-based sclerotherapy or EBL) for the immediate control of bleeding (93.9% vs. 79.5%, *p* = 0.03) [15]. 

Glue embolism is associated with unfavorable clinical outcomes despite being rare. The largest study on the subject reported clinically manifested pulmonary embolism in 0.7% of cases [16]. The real incidence of glue emboli may be significantly higher, but the lack of prominent symptoms often hinders diagnosis.

In the presented case, ECI was the preferred first-line treatment according to existing guidelines. It outlines certain pitfalls in applying the procedure, particularly the inability to verify the presence of complete variceal occlusion. The anticipated risk of glue embolism generally leads to an insufficient volume of glue injection, resulting in incomplete variceal obliteration.

Currently, there are no clear recommendations on the optimal secondary prophylaxis for GVB. In general, TIPS and BRTO are considered viable options with comparable clinical outcomes [8]. TIPS is an effective modality for managing EVs. However, GV’s tendency to bleed at lower portal pressures may lead to a rebleeding rate as high as 50% after TIPS [17]. 

In the current paper, the patient had absolute contraindications for TIPS [18]. BRTO was not discussed due to a lack of expertise. Such a decision would probably be inappropriate considering the patient’s prior history of EV bleeding. We established that BRTO leads to EV progression in 30–35% of cases [19]. Alternative modalities for secondary prophylaxis were evaluated after the patient was considered ineligible for radiological interventions. 

Applying EUS-CIT as a secondary prophylaxis for GVB was extensively investigated in a recent meta-analysis by Chandan et al. [20]. They established that EUS-CIT is highly effective for both primary and secondary prophylaxis of GVB, achieving 95.4% and 88.7% treatment efficiency, respectively. The pooled rate of GV obliteration was 87%, with an 11.9% complication rate. In only 7.7% of patients, salvage therapy (mainly TIPS) was needed. Comparing EIT and EUS-CIT, EUS-CIT had an improved rebleeding rate when compared to EIT.

The EUS-CIT technique includes the initial puncture of the gastric varices under endosonographic guidance with an FNA needle. In our report, we used a 19 Ga needle, which was used in most cases and described in the literature [11,14,21,22,23,24]. The rationale is that 19 Ga needles can accommodate 0.035″ embolization coils, which are the most common choice. 

There are two modalities for variceal puncture reported in the literature. Binmoeller et al. used the so-called “transcrural” access to describe the technique. This approach includes puncturing the varix through the distal esophageal and crus of the diaphragm. Afterward, the “transgastric” approach was described, which consists of direct puncturing of the varix through an overlying stomach mucosa. Although the transgastric approach is associated with a higher risk of bleeding and technical difficulty, it may be preferable when there is a large EV present. Comparative studies failed to achieve significant differences between the clinical outcomes of the two techniques. In the current case, we used a transgastric approach to visualize the gastric vessels through the esophagus, which was inadequate. 

Another aspect under debate is the optimal choice of target vessel. Romero-Castro et al. speculated that selecting perforated vessels would induce better GV occlusion while reducing the total glue volume needed and, hence, the risk of systemic embolization [25]. On the other hand, selecting a perforating vessel carries a risk of inadvertent injection into an efferent vessel or peri-gastric branch of the splenic vein, which has potentially disastrous consequences. To avoid these potential pitfalls, we decided to inject directly into the varix, an agreed-upon approach in more recent studies [14,23].

The standard approach to coil introduction involves positioning the puncture needle at a distance from the opposite wall of the vessel to reduce perforation risk [26]. The diameter of the coils should be 20–30% larger than the diameter of the vessel to preclude migration [26]. We followed those recommendations with the largest diameter of the varix being 10 mm. We also used two 14 mm/7 cm 0.035″ embolization coils. 

Regarding the glue injection technique, there are certain discrepancies between different authors. Recent AGA guidelines warn against using plant-based oils in combination with cyanoacrylate due to increased embolization risk [8]. Binmoeller et al. used 2-octyl-cyanocrylate, which has a significantly longer polymerization time [14]. Based on existing guidelines and our own experience, we opted to inject “pure” N-butyl cyanoacrylate. Rinsing the needle properly is essential to avoid premature polymerization. We used glucose solution for rinsing based on our experience with ECI, suggesting that 5% glucose solution slightly delays the polymerization of cyanoacrylate. 

After the procedure was completed, the needle was withdrawn from the varix, but not from the endoscope. The latter was still in the patient. Glue-induced damage from endoscopes might be irreversible, so every effort should be made to avoid it. 

EBL was conducted during a follow-up endoscopy at week 4. These results align with existing guidelines for pursuing the complete eradication of EVs [9].

## 4. Conclusions

Our initial experience with EUS-CIT suggests that it is a viable alternative to ECI therapy after achieving excellent variceal occlusion and minimizing the risk of systemic embolization. It can be an effective secondary prophylaxis for recurrent GVB. EUS-CIT is a valid alternative for patients with contraindications for radiological interventions (TIPS and/or BRTO) and may be considered in the presence of adequate expertise.

## Figures and Tables

**Table 1:** Sarin’s classification of gastric varices.

Sarin’s Type of Gastric Varix	Description
Gastro-oesophageal varices type 1 (GOV type I)	Extension of the esophageal varices along the cardia toward the lesser curvature (straight course)
Gastro-oesophageal varices type 2 (GOV type II)	Extension of the esophageal varices along the cardia toward the greater curvature (more tortuous course)
Isolated gastric varices type 1 (IGV type 1)	Gastric varices in the fundus (complex and tortuous) in the absence of esophageal varices
Isolated gastric varices type 2 (IGV type 2)	Gastric varices outside the fundus (body, antrum, pylorus) in the absence of esophageal varices

**Table 2:** Overview of liver disease course.

Disease Course	Ascites	Encephalopathy	Bil. (µmol/L)	INR	Alb. (g/L)	Creatinine (µmol/L)	Na (mmol/L)	Dialysis	CTP Class	MELD-Na Score
Initial presentation	Mild	No	18.4	1.2	29	114	140	No	B	11
EVB episode	Mild	No	44.2	1.9	24	87	136	No	C	18
GVB episode I	Mild	No	16.3	2.0	28	84	133	No	B	17
GVB episode II	Moderate	No	20.3	2.0	24	67	144	No	B	15
EUS-CIT	Mild	No	36.2	1.36	29	90	140	No	B	13
Follow-up	Moderate	No	40.6	1.30	34	91	139	No	B	13

EVB—esophageal variceal bleeding; GVB—gastric variceal bleeding, EUS-CIT—endoscopic ultrasound combined injection therapy, CTP—Child–Turcotte–Pugh, MELD-Na—Model of End-stage Liver Disease–Sodium. INR—international normalized ratio.

## Data Availability

Data are available upon request due to privacy restrictions.

## Abbreviations

VB	variceal bleeding
GOV	gastro-esophageal varices
GV	gastric varices
EVs	esophageal varices
GVB	gastric variceal bleeding
IGV	isolated gastric varices
EBL	endoscopic band ligation
ECI	endoscopic cyanoacrylate injection
AEs	adverse events
EUS	endoscopic ultrasound
TIPS	transjugular intrahepatic portosystemic shunt
BRTO	balloon occlusive retrograde transvenous obliteration
EUS-CIT	endoscopic ultrasound combined with injection therapy
INR	international normalized ratio
FNA	fine needle aspiration

## Appendix A

**Table A1:** The Child–Turcotte–Pugh (CTP) classification of liver cirrhosis [27].

Parameter	Points Assigned		
	1	2	3
Ascites	Absent	Mild to moderate (diuretic responsiveness)	Severe (diuretic refractory)
Hepatic encephalopaty	None	Grades 1–2	Grades 3–4
Bilirubin (µmol/L)	<34.2	34.2–51.3	>51.3
INR	<1.7	1.7–2.3	>2.3
Albumin (g/L)	>35	28–35	<28

CPT classification; Child A—5–6 points—well-compensated; Child B—7–9 points—significant functional compromise; Child C—10–15 points—decompensated.

**Table A2:** Baveno classification of esophageal varices [3].

Size	Description
Small	Minimally elevated varices above the esophageal mucosa surface
Medium	Tortuous varices occupying less than one third of the esophageal surface
Large	Varices occupying more than one-third of the esophageal surface
